# Dynamic expression of homeostatic ion channels in differentiated cortical astrocytes in vitro

**DOI:** 10.1007/s00424-021-02627-x

**Published:** 2021-11-04

**Authors:** Francesco Formaggio, Martina Fazzina, Raúl Estévez, Marco Caprini, Stefano Ferroni

**Affiliations:** 1 Department of Pharmacy and Biotechnology, University of Bologna, Via San Donato 19/2, 40127 Bologna, Italy; 2 Present Address: Department for Life Quality Studies, University of Bologna, Rimini, Italy; 3 Departament de Ciències Fisiològiques, IDIBELL-Institute of Neurosciences, Universitat de Barcelona, Barcelona, Spain; 4 Centro de Investigación Biomédica en Red Sobre Enfermedades Raras (CIBERER), Instituto de Salud Carlos III, Madrid, Spain

**Keywords:** Cultured astrocytes, Ion channels, K^+^ channel, Cl^−^ channel, Brain homeostasis

## Abstract

The capacity of astrocytes to adapt their biochemical and functional features upon physiological and pathological stimuli is a fundamental property at the basis of their ability to regulate the homeostasis of the central nervous system (CNS). It is well known that in primary cultured astrocytes, the expression of plasma membrane ion channels and transporters involved in homeostatic tasks does not closely reflect the pattern observed in vivo. The individuation of culture conditions that promote the expression of the ion channel array found in vivo is crucial when aiming at investigating the mechanisms underlying their dynamics upon various physiological and pathological stimuli. A chemically defined medium containing growth factors and hormones (G5) was previously shown to induce the growth, differentiation, and maturation of primary cultured astrocytes. Here we report that under these culture conditions, rat cortical astrocytes undergo robust morphological changes acquiring a multi-branched phenotype, which develops gradually during the 2-week period of culturing. The shape changes were paralleled by variations in passive membrane properties and background conductance owing to the differential temporal development of inwardly rectifying chloride (Cl^−^) and potassium (K^+^) currents. Confocal and immunoblot analyses showed that morphologically differentiated astrocytes displayed a large increase in the expression of the inward rectifier Cl^−^ and K^+^ channels ClC-2 and Kir4.1, respectively, which are relevant ion channels in vivo. Finally, they exhibited a large diminution of the intermediate filaments glial fibrillary acidic protein (GFAP) and vimentin which are upregulated in reactive astrocytes in vivo. Taken together the data indicate that long-term culturing of cortical astrocytes in this chemical-defined medium promotes a quiescent functional phenotype. This culture model could aid to address the regulation of ion channel expression involved in CNS homeostasis in response to physiological and pathological challenges.

**Supplementary Information:**

The online version contains supplementary material available at 10.1007/s00424-021-02627-x.

## Introduction

In the last decades, a large body of evidence have accumulated showing that in the central nervous system (CNS), astroglial cells are actively involved in the maintenance of neuronal health through various mechanisms including neuronal metabolic and trophic supports, the homeostatic regulation of the perineuronal milieu, and neurovascular support [82]. The ability of astrocytes to influence the physiological response of neuronal circuits is also corroborated by the wealth of information reporting that, as consequence of a variety of acute and chronic pathological conditions, astrocytes can become activated (reactive) and respond to various insults through alterations of morphological, molecular, and biochemical processes [91].

Fully functional astrocytes in vivo are endowed with a variety of ion channels and transporters some of which have unequivocally been shown to have housekeeping homeostatic roles [61, 83]. Astrocytes express a large background potassium (K^+^) conductance carried mainly by the weakly inward-rectifier K^+^ channel 4.1 (Kir4.1); this K^+^ conductance sets the very negative membrane potential, contributes to the maintenance of the extracellular K^+^ homeostasis, and regulates the efficacy of glutamate uptake [6, 15, 55, 56]. Kir4.1 is strongly developmentally regulated being virtually absent at birth and showing a sharp increment in expression at 10 days postnatal [75]. Furthermore, Kir4.1 channel is also downregulated under various pathological conditions [5, 26, 44, 58, 67]. Of note, when studied in culture conditions, Kir channels are not functionally expressed [1, 3, 21]. Hence the identification of culture conditions that can more closely reflect the in vivo situation is mandatory to address the molecular mechanisms that contribute to the development and maintenance of a mature functional phenotype and those that induce reactive astrogliosis in which the expression of ion channels is altered.

We previously reported that a Kir conductance and an inward rectifier chloride (Cl^−^) current were observed when cultured astrocytes were exposed for long term to conditions that elevate cytosolic cyclic AMP, which induces their morphological differentiation from flat polygonal to a phenotype with long thick processes [21]. Other studies reported that cultured rodent astrocytes, grown in the presence of a morphologically differentiating supplement (G5) containing growth factors and hormones [50], displayed the upregulation of glutamate uptake through mechanisms mediated, at least partially, by augmented levels of the sodium-dependent glutamate transporters GLT-1 and GLAST [7, 27, 85]. There is also indication that G5 augmented the saxitoxin-sensitive voltage-gated sodium (Na^+^) channels [90]. At variance, cultured astrocytes from rat corpus callosum exposed to G5 did not evidence any significant alterations in K^+^ conductance [72].

Because of these variable effects of G5 supplementation on the expression of plasma membrane homeostatic proteins in this work, we sought to determine whether the morphological differentiation of primary astrocytes induced by adding G5 to the culture medium devoid of fetal bovine serum (FBS) was associated to the expression of homeostatic ion channels which are not present in conventionally cultured astrocytes. Our results show that rat cortical astrocytes cultured for long term in G5-containing medium acquired a differentiated morphological phenotype and displayed K^+^ and Cl^−^ conductance which are found in mature astrocytes. Functional and molecular analyses indicate that G5-treated astrocytes exhibited an increase in Kir4.1 and ClC-2 proteins. This culture protocol could be suitable to address the dynamic mechanisms that influence the expression of functionally relevant ion channels in long-term cultured astrocytes under physiological and pathological conditions.

## Materials and methods

### Preparation of primary cultures of neonatal rat cortical astrocytes

All the experiments were performed according to the Italian law on protection of laboratory animals, with the approval of bioethical committees of the University of Bologna (AEDB0.2) and of the Ministry of Health (protocol number 83/2017-PR) and under the supervision of the veterinary commission for animal care and comfort at the University of Bologna.

Primary cultures of cortical astrocytes from newborn (1–2 days) Sprague–Dawley breeding pairs (Charles River, Italy) were obtained according to the standard method [47] with some modifications [21] to obtain highly purified cultures composed of more than 95% astrocytes. Cultured flasks were maintained in DMEM containing GlutaMAX™-I and 4.5 g/L D-glucose, with 10% of heat-inactivated fetal bovine serum (FBS) and penicillin–streptomycin (100 U/mL and 100 μg/mL respectively). All products were from Gibco-BRL.

### G5 treatment of primary cortical astrocytes

Confluent astroglial monolayers were enzymatically detached using trypsin (Gibco-BRL) and plated onto Petri dishes at low density (see below). Two days after, FBS was reduced to 3%, and G5 supplement (Sigma-Aldrich) was added (10 μL/mL) to the culture medium. G5 is a chemical-defined supplement based on a formulation composed of growth factors, hormones, and micronutrients that induces the morphological differentiation of cultured astrocytes and lowers proliferation rate [50]. After 2 days of culturing in medium containing 3% FBS and G5, at the next medium change FBS was removed and G5 was present for the remaining period of culturing up to 16 days (days of treatment, DOT). G5 was freshly added every 4-5 DOT when changing the medium (Table 1). Untreated astrocytes were maintained in the same culture conditions in the absence of G5.

**Table 1:**
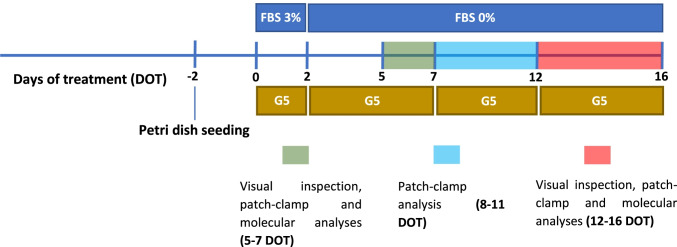
Timeline of G5 treatment and data collection

### Electrophysiology

For electrophysiological experiments, untreated astrocytes were plated in 35-mm Petri dishes at a density of 24,000 cells per dish, whereas, to avoid cell–cell contact, highly branched G5-treated astrocytes were plated at 8,000 or 12,000 cells per dish. Whole-cell patch-clamp experiments were carried out as previously described [20, 21]. During each day of experiment, recordings were performed alternating measurements in G5-treated and untreated (ctrl) astrocytes. Petri dishes were mounted on the stage of an inverted microscope equipped with phase-contrast optics (Nikon Diaphot). Patch-clamp pipettes were prepared from borosilicate glass capillaries to have a tip resistance of 2–4 MΩ when filled with the standard intracellular solution. Membrane currents were amplified with an EPC-7 amplifier (List Electronic, Darmstadt Germany) and low-pass filtered at 3 kHz before acquisition. Stimulation and analysis were performed with pClamp 6 software (Axon Instrument, Foster City, CA, USA) and Origin 6.0 (MicroCal, Northampton, MA, USA). All experiments were carried out at room temperature (20–24 °C). Series resistance values were below 10–12 MΩ and were corrected for by 40–60% with the analog circuit of the amplifier. Cell capacitance of the recorded cell was obtained by the amplifier reading of the capacitive transient cancelation.

The standard external (bath) solution was (mM) 140 NaCl, 4 KCl, 2 MgCl_2_, 2 CaCl_2_, 10 HEPES 5 glucose, pH 7.4 adjusted with NaOH, and osmolarity ~ 310 mOsm corrected with mannitol. The standard intracellular (pipette) solution was composed of (mM) 144 KCl, 2 MgCl_2_, 5 EGTA, 10 HEPES, pH 7.2 adjusted KOH, and osmolarity ~ 290 mOsm with mannitol. When using external solution with different ionic compositions, salts were replaced equimolarly. To isolate Cl^−^ currents, the external solution used was (mM) 120 CsCl, 2 MgCl_2_, 2 CaCl_2_, 10 2‐[Tris (hydroxymethyl)‐methylamino]‐ethanesulfonic acid (TES), 5 glucose, pH 7.2 adjusted with CsOH, and osmolarity ∼310 mOsm with mannitol. The pipette solution was composed of (mM) 125 CsCl, 2 MgCl_2_, 5 EGTA, 10 TES, pH 7.2 adjusted with CsOH, and osmolarity ∼290 mOsm with mannitol. Changes of solutions around the recorded cells were performed by using a gravity-driven, local perfusion system at a flow rate of ~ 200 μL/min and positioned within ~ 100 μm of the recorded cell.

### Western blot

The primary and secondary antibodies used are listed in Table 2. Astrocytes were seeded in 60-mm Petri dishes, and cell lysates from G5-treated and untreated astrocytes were obtained at 5–7 and 12–16 DOT. Cells were lysed with 200 μL of RIPA buffer and quantified with the Bradford method, as previously described [24]. Proteins were separated in a 10–12% SDS–polyacrylamide, blotted into PVDF membrane, and incubated 1 h at room temperature (RT) in blocking solution made of PBST 0.1% containing 5% BSA (Sigma-Aldrich). Membranes were incubated with primary antibodies overnight at 4 °C in PBST 0.1% containing 1% BSA. The following day, they were probed with IgG horseradish peroxidase‐conjugated secondary antibodies and developed with the enhancing chemiluminescence detection system (Santa Cruz Biotechnology or Cyanagen–Westar ηC ultra 2.0). Blots were visualized with the ChemiDoc^TM^ (Bio-Rad) imaging system, and densitometric analysis was performed using the Image Lab 6.0 software. Immunoreactive bands were normalized against β -actin levels.

**Table 2:** List of antibodies used for western blot analysis

Primary antibodies	Mouse anti-Kir4.1 (Santa-Cruz Biotechnology, sc-293252) (1:500)
	Mouse anti-GFAP (Santa-Cruz Biotechnology, sc-33673) (1:1000)
	Rabbit anti-β-actin (Sigma-Aldrich, A2066) (1:1000)
	Rabbit anti-ClC-2 (custom-made provided by R. Estévez) (1:500)
	Mouse anti-vimentin (Santa-Cruz Biotechnology, sc-6260) (1: 1500)
	Mouse anti-GLT-1 (Santa-Cruz Biotechnology, sc-365634) (1:500)
Secondary antibodies	Goat anti-mouse-HRP (Sigma-Aldrich, A4416) (1:5000 for all primary antibodies)
	Goat anti-rabbit-HRP (Sigma-Aldrich, A12-348) (1:5000 for anti-ClC-2, 1:10000 for anti-β-actin)

### Immunofluorescence

The primary and secondary antibodies used are listed in Table 3. For immunofluorescence experiments, cortical astrocytes were plated on glass coverslips coated with poly-D-lysine (Sigma-Aldrich) (100 μg/mL) at a concentration of 5000 cells/coverslip for both untreated and G5-treated astrocytes. Immunocytochemical analyses were performed as previously described [24]. Briefly, primary cultured astrocytes were washed twice in PBS, fixed with 4% paraformaldehyde (Sigma-Aldrich) at RT for 10 min, and washed again twice in PBS for 10 min. To block non-specific staining, astrocytes were incubated for 1 h at RT in PBS containing 5% BSA and 0.05% Triton X-100 (Sigma-Aldrich). After blocking, specimens were probed overnight at 4 °C with primary antibodies, which were diluted in PBS containing 1% BSA and 0.05% Triton X-100. Coverslips were washed 3 times for 20 min in PBS at RT and incubated 2 h with secondary antibodies diluted in PBS containing 1% BSA and 0.05% Triton X-100. Coverslips were finally washed twice for 5 min and mounted onto poly-lysine-coated slides (Menzel-Gläser Superfrost, Thermo Scientific) with Fluoromount-G mounting medium (Sigma-Aldrich). Single‐plane confocal immunofluorescence images were acquired with an inverted laser scanning confocal microscope (Nikon D-Eclipse C1). To assess staining specificity, astrocytes were also processed in the absence of primary antibodies. All secondary antibodies were conjugated with Cyanine 2/3.

**Table 3:** List of antibodies used for immunofluorescence analysis

Primary antibodies	• Rabbit anti-Kir4.1 (Alomone, APC-035) (1:200)
	• Rabbit anti-ClC-2 (provided by Prof. R. Estévez) (1:200)
	• Chicken anti-GFAP (BioLegend, Poly28294) (1:500)
Secondary antibodies	• Cy-2 donkey anti-rabbit (Jackson ImmunoResearch, 711–225-152) (1:400)
	• Cy-3 donkey anti-chicken (Jackson ImmunoResearch, 703–165-155) (1:400)

### Statistical analysis

Data are presented as mean ± standard error or box plots. The D’Agostino-Pearson omnibus test was used to detect consistency of normal distribution. The Grubbs’s test was used to detect outliers at the 95% confidence level. The significance was determined with the Student’s *t*-test (paired and unpaired, two-tailed), one-way ANOVA, or Kruskal–Wallis test as appropriate by using GraphPad Prism software (GraphPad Software, Inc., San Diego, CA, USA). A *p*-value < 0.05 was considered statistically significant.

## Results

### Time-dependent morphological differentiation of G5-treated primary cortical astrocytes

**Fig. 1:**
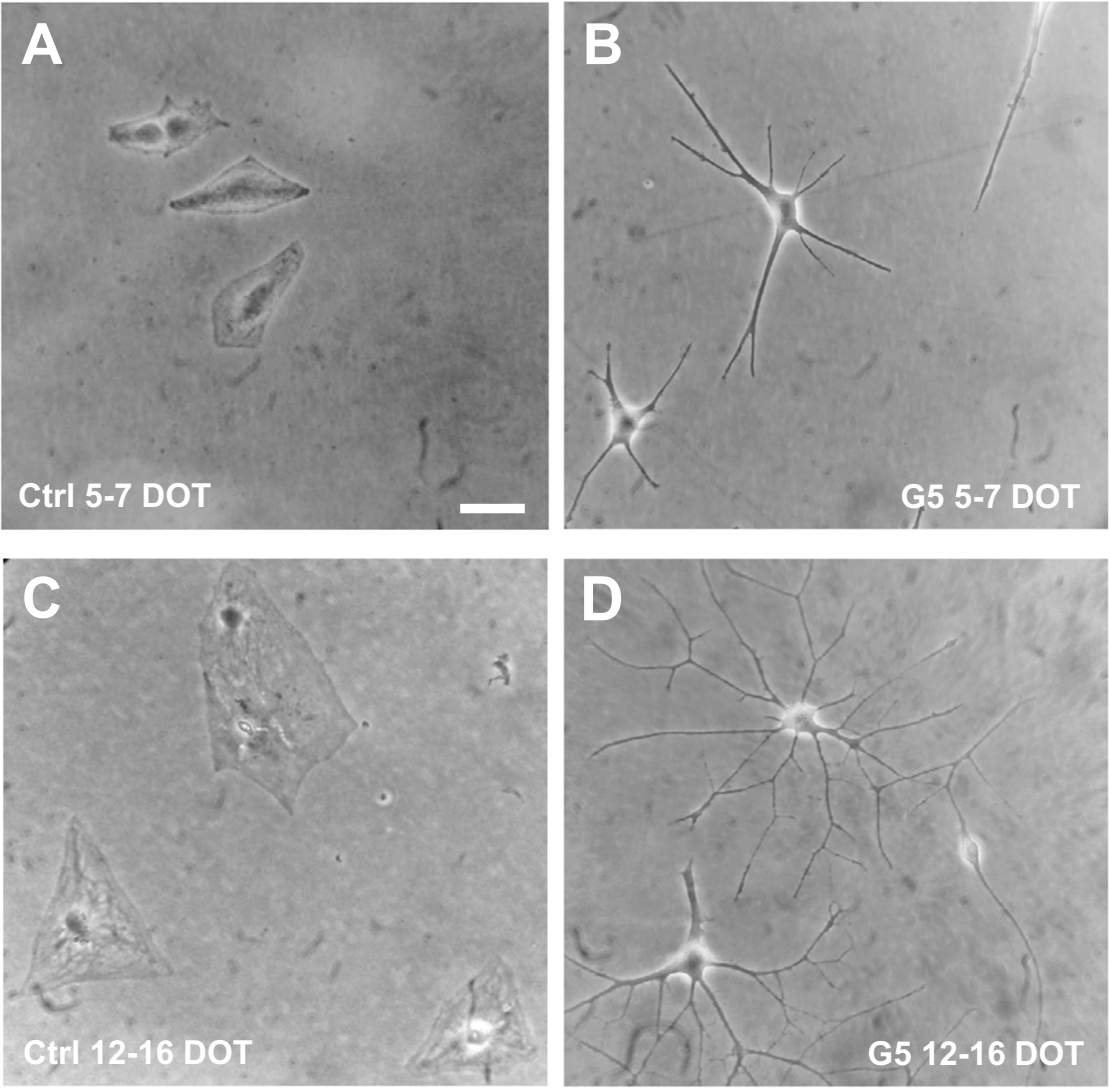
Time-dependent changes of morphological phenotype in G5-treated primary cultured rat cortical astrocytes. Phase-contrast micrographs of low-density replated astrocytes in FBS-free culture conditions in the absence (ctrl) and presence of G5 supplement for various days of treatment (DOT). **A** Untreated astrocytes at 5–7 DOT display the typical epithelioid shape. **B** Subconfluent astrocytes exposed to G5 for 5–7 DOT show few polarized processes elongating from an irregular cell body. **C** Untreated subconfluent astrocytes at 12–16 DOT have a flat, epithelioid morphology. **D** Low-density G5**-**treated astrocytes at 12–16 DOT develop multi-branched processes radially departing from retracted cell bodies. Scale bar: 20 µm

Previous works showed that few days of exposure of cultured astrocytes to growth medium supplemented with G5 resulted in shape changes characterized by a shift from an epithelioid to a process-bearing morphology and an increase in glutamate uptake [86, 87]. Since we were interested to individuate other functional changes which paralleled the G5 effect on glutamate dynamics, cultured cortical astrocytes were challenged with G5 for a period of up to 16 DOT. Initial analysis was devoted to determining the long-term effect of G5 on the morphology of astrocytes cultured in the absence of FBS. The results show that after 5–7 and 12–16 days in FBS-free control medium (ctrl), astrocytes replated at low density exhibited the epithelioid shape typical of undifferentiated primary cultured rat cortical astrocytes [21] (Fig. 1A, C). Cultured astrocytes exposed to G5 for 5–7 DOT displayed a cell shape with few polarized processes elongating from an irregular cell body (Fig. 1B) and acquired a multi-branched phenotype radially departing from a retracted cell body at 12–16 DOT with G5 (Fig. 1D), These results indicate that the growth of cultured cortical astrocytes in a G5-containing chemical-defined medium promotes shape changes toward a process-bearing phenotype that develops gradually during the 16 DOT.

### Electrical membrane properties of G5-treated primary cortical astrocytes

**Fig. 2:**
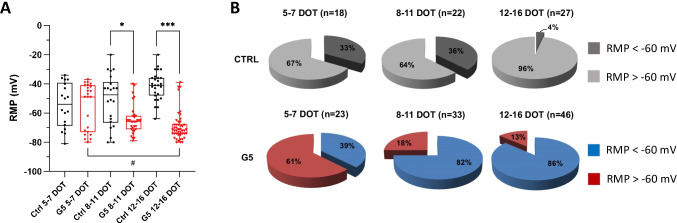
Time-dependent variations in resting membrane potentials in G5-treated primary cortical astrocytes. **A **Box plots of resting membrane potential (RMP) values depicting the median and the interquartile range in untreated (ctrl, black) and G5-treated (red) astrocytes at different DOT windows. Untreated astrocytes display a median RMP that remains stable at ~ −50 mV throughout the whole period of subculturing (5–7 DOT, *n*=18; 8–11 DOT, *n*=22; 12–16 DOT, *n*=27).In G5-treated astrocytes, the median RMP is significantly more hyperpolarized after 8–11 DOT compared to untreated astrocytes (5–7 DOT, *n*=23; 8–11 DOT, *n*=32; 12–16 DOT, *n*=51). *p<0.05, ***p<0.001, and #*p*<0.05 with Kruskal-Wallis test. Where not indicated, the group differences were non-significant. **B** Pie graphs of the percentage of astrocytes with RMPs more negative (<) and more positive (>) of −60 mV in untreated (ctrl) and G5-treated cells. The value of −60 mV depicts the value of RMP separating the two populations of astrocytes described by the binomial distribution of RMPs in G5-treated astrocytes (Fig. S1). *N* is the number of cells analyzed in each condition at different DOT windows

We next addressed whether the dynamic changes in cell shape observed under these culture conditions were paralleled by alterations in electrical membrane properties. Experiments were carried out with the patch-clamp technique using standard intra- and extracellular solutions. We initially compared the resting membrane potential (RMP) of cortical astrocytes cultured in the absence (ctrl) and presence of G5 measured at various time of G5 treatment. Whereas RMP values were normally distributed in untreated astrocytes at all DOT windows, those of G5-treated astrocytes followed a bimodal distribution (Fig. S1). The non-parametric analysis (Fig. 2A and Table 4 of S1) shows that in untreated astrocytes (ctrl), RMPs were not significantly different at all DOT windows. RMP values in untreated and G5-treated astrocytes were not different at 5–7 DOT but were significantly more hyperpolarized in 8–11 and 12–16 DOT G5-treated astrocytes. Moreover, RMPs become more negative with prolonged time of G5 exposure. Because the hyperpolarized RMPs of G5-treated astrocytes could be due to the presence of astrocyte subpopulations [39, 48, 93] with different sensitivity to G5 challenge, we next performed a stratified analysis to assess whether the time of exposure to G5 affected the percentage of cells with RMP higher and lower than a threshold value of − 60 mV. This value was chosen because in the bimodal distribution it separated the two RMP populations in G5 astrocytes. The results indicate that whereas in untreated astrocytes the number of cells displaying RMPs more negative than − 60 mV was diminished at 12–16 DOT (Fig. 2B, upper), in G5-treated cells, there was a positive correlation between time of exposure to G5 and percentage of astrocytes with RMPs more negative than − 60 mV (Fig. 2B, lower).

**Fig. 3:**
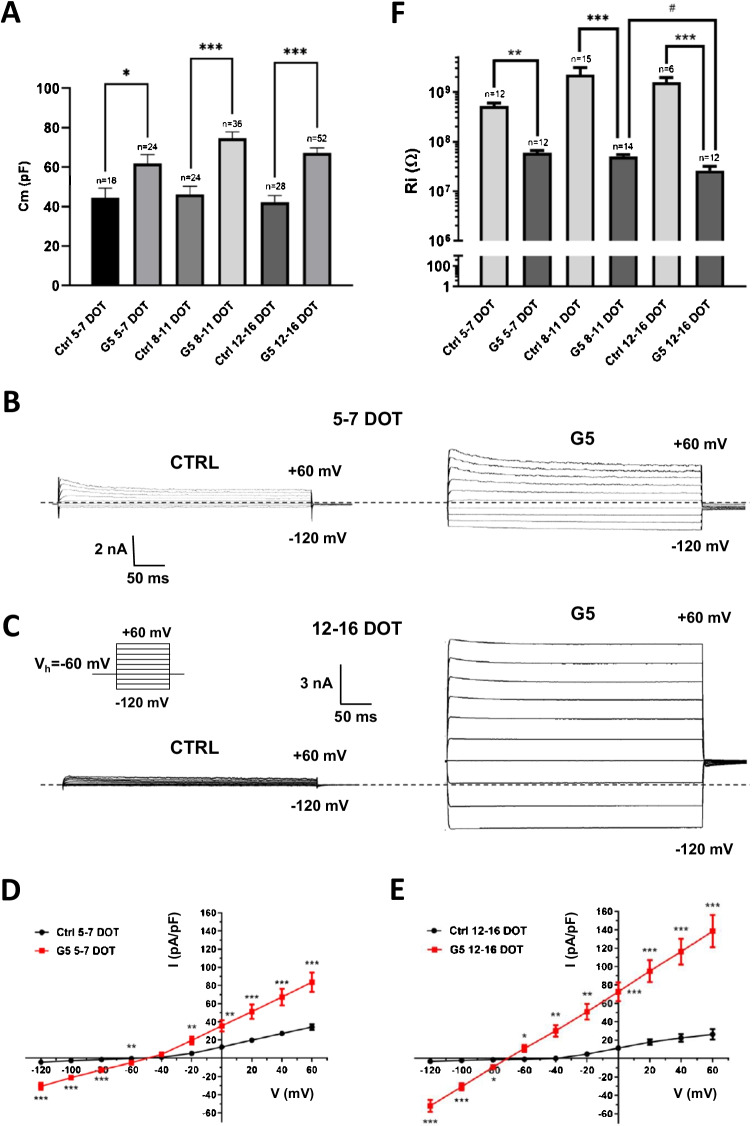
Time-dependent effects of G5 challenge on whole-cell membrane conductance of primary cortical astrocytes. **A **Bar graph of mean membrane capacitance (Cm) values in untreated (ctrl) and G5-treated astrocytes at the various DOT windows. *p<0.05 and ***p<0.001 with one-way ANOVA followed by Bonferroni's multiple comparisons test. **B **Representative current families elicited with a family of 400-ms voltage steps from a holding potential (*V*_*h*_) of −60 mV and steps from −120 to +60 mV in 20-mV increments (inset of C) in an 5–7 DOT untreated (ctrl) astrocyte (left) and in an astrocyte grown in G5 for the same DOT (right). **C** Representative current families at 12–16 DOT untreated (left) and G5-treated astrocytes (right). Dashed lines in B and C are the zero-current levels. **D** Current-voltage relationship (I-V) of mean steady-state current densities from 5 to 7 DOT untreated (ctrl, black, *n*=13) and 5–7 DOT G5 astrocytes (red, *n*=12). **E** I-V plot of 12–16 DOT untreated astrocytes (ctrl, black, *n*=6) and upon 12–16 DOT with G5 (red, *n*=11) *p<0.05, **p<0.01, and ***p<0.001 with unpaired Student’s *t*-test. **F** Semi-logarithmic bar graph of the mean values of the input resistance in the two conditions at different DOT windows. *p<0.05, **p<0.01, ***p<0.001, and #*p*<0.05 with one-way ANOVA followed by Bonferroni’s multiple comparisons test

We next examined whether the gradual hyperpolarizing shift in RMP observed in the G5-treated, morphologically differentiated astrocytes was associated to changes in the other passive membrane properties. The values of cell membrane capacitance (Cm) of untreated astrocytes at all DOT windows were not significantly different. Compared to untreated astrocytes, those cultured in the presence of G5 revealed an increase in Cm at all DOT (Fig. 3A). We next determined the membrane resistance associated to the background membrane conductance. To unravel the background conductance, astrocytes were voltage clamped at the holding potential (*V*_*h*_) of − 60 mV, and families of 400-ms-long voltage steps of 20 mV increments were delivered from − 120 to + 60 mV (Fig. 3C, inset). Untreated astrocytes at all DOT displayed only outwardly rectifying, non-inactivating currents at membrane potentials more positive than − 40 mV (Fig. 3B and C, left). Their voltage-dependent kinetics (Fig. 3E, F) and the partial inhibition by extracellular administration of 10 mM TEA (data not shown) strongly suggest that they were mainly mediated by voltage-gated K^+^ channels [21]. G5-treated astrocytes at 5–7 DOT exhibited an increase in membrane conductance (Fig. 3B, right). Both positive and negative fast activating, non-inactivating membrane currents were elicited in the entire range of voltage stimulation. The currents had a quasi-linear current–voltage (I-V) profile (Fig. 3D) and changed polarity (*V*_rev_) at ~  − 50 mV. At 12–16 DOT, larger quasi-instantaneous, non-inactivating currents were evoked both at positive and negative membrane potentials (Fig. 3C, right). Currents had *V*_rev_ at ~  − 70 mV and exhibited an ohmic behavior (Fig. 3E). The increase in membrane conductance upon time of exposure to G5 was also evident when comparing the I-V relationships of G5-treated astrocytes at 5–7 and 12–16 DOT (Fig. S2). The analysis of the input resistance (Ri) from the linear portion of the I-V curves at negative voltages measuring the instantaneous current from − 60 to − 80 mV revealed that compared to untreated astrocytes, G5 exposure caused a significant decrement of Ri at all DOT windows (Fig. 3F). A decrease in Ri was also observed when comparing G5 astrocytes at 8–11 and 12–16 DOT.

Taken together, these results show that compared to control astrocytes, those morphologically differentiated upon long-term exposure to the chemical-defined medium containing G5 display an augment of the background membrane conductance mediated by the time-dependent increase in different membrane currents.

### Differential pharmacological modulation of membrane conductance expressed by G5-treated primary cortical astrocytes

Since the experiments above suggested the differential expression of membrane currents that contribute to the background conductance depending on the period of astrocyte exposure to the G5-containing medium, we next addressed their pharmacological sensitivity. Previous work showed that cortical astrocytes morphologically differentiated upon long-term exposure to a membrane-permeable analog of cyclic-AMP (dibutyryl-cAMP, dBcAMP) expressed inward rectifier K^+^ and Cl^−^ currents that contribute to set the RMP and are specifically inhibited by extracellular exposure to micromolar concentrations of barium (Ba^2+^) and cadmium (Cd^2+^), respectively [21]. We hence addressed the ability of these ions to depress the currents expressed by G5-treated cultured astrocytes.

**Fig. 4:**
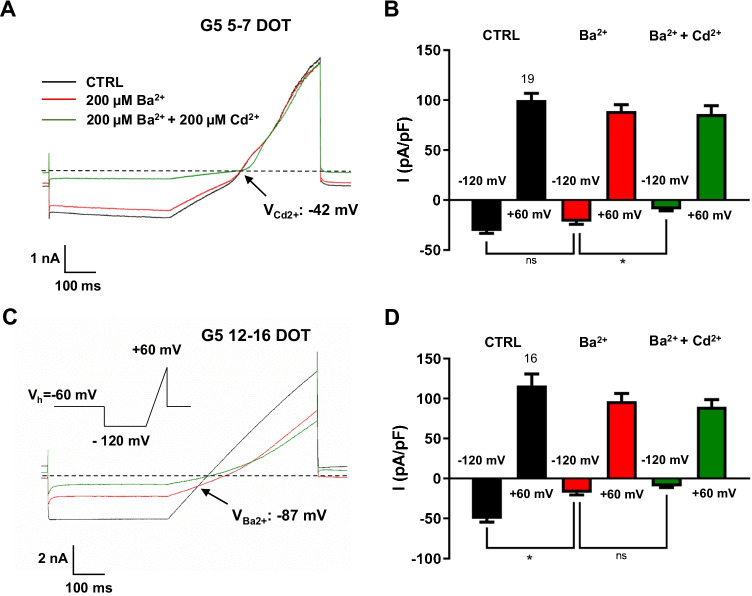
Time-dependent differential pharmacological sensitivity of whole-cell membrane conductance in G5-treated primary cortical astrocytes. **A **Representative currents evoked in a 5–7 DOT G5 astrocyte with a ramp protocol (inset of C) stepping from a *V*_*h*_ of −60 to −120 mV for 400 ms before applying a depolarizing ramp (180 mV/500 ms). Currents are those remaining stable for at least 2 min in control conditions (ctrl, black trace), following the maximal effect of barium administration (Ba^2+^, 200 µM, red trace) and upon the subsequent addition of cadmium (Cd^2+^, 200 µM) to the Ba^2+^-containing solution (green trace). The voltage intercept (V_Cd2+_) of the currents before and after addition of Cd^2+^ was near the zero-current potential. Dashed line is the zero-current level. The RMP of this astrocyte was −44 mV. **B** The bar graph of the quantitative analysis of the current densities at +60 mV and −120 mV in the absence and presence of Ba^2+^ and Ba^2+^ + Cd^2+^ depicts a significant current diminution only at −120 mV upon Cd^2+^ administration. *p<0.05 with one-way ANOVA followed by Bonferroni’s multiple comparisons test. **C** Representative ramp current from a 12-16 DOT G5 astrocyte with a RMP of −76 mV and elicited with the same protocol as in A. The voltage intercept (*V*_Ba2+_) of the ramp currents before and after addition of Ba^2+^ is close to the Nernst equilibrium potential for potassium (K^+^) under the experimental conditions used. Dashed line is the zero-current level. **D** The bar graph of the quantitative analysis of the current densities at +60 mV and −120 mV in the absence and presence of Ba^2+^ and Ba^2+^ + Cd^2+^ shows that a significant current attenuation was observed only at −120 mV. *p<0.05 with one-way ANOVA followed by Bonferroni’s multiple comparisons test. Numbers above black bars in B and D depict the number of cells in each condition. Ns identifies non-significant differences

In Fig. 4 are shown the differential Ba^2+^ and Cd^2+^ sensitivities of the background conductance in astrocytes analyzed at the two most distant DOT windows. In 5–7 DOT G5 astrocytes, both positive and negative ramp currents elicited from − 120 to + 60 mV were not affected by extracellular Ba^2+^ (200 μM). By contrast negative currents were inhibited by subsequent co-administration of Cd^2+^ (200 μM) and Ba^2+^ (Fig. 4A, B). The voltage intercept of ramp currents before and after Ba^2+^ and Cd^2+^ co-administration was near the zero-current level. The same stimulation protocol revealed that in 12–16 DOT G5 astrocytes, Ba^2+^ caused a depression of the ramp current (Fig. 4C), which, however, was significant only at negative voltages and was not further attenuated by co-application of Cd^2+^ and Ba^2+^ (Fig. 4D). The voltage intercept of ramp currents before and after Ba^2+^ administration was at ~  − 85 mV strongly suggesting that in these astrocytes, the resting conductance was mediated by a Ba^2+^-sensitive K^+^ conductance. Overall, in this analysis, G5-treated astrocytes at 5–7 DOT with RMPs more positive than − 60 mV (12 of 15 astrocytes) displayed the prevalence of Cd^2+^-sensitive current with a non-significant contribution of the Ba^2+^-sensitive K^+^ current. The opposite was observed in 12–16 DOT G5 astrocytes which had RMPs more negative than − 60 mV (26 of 31 astrocytes). Altogether these findings confirm that astrocytes morphologically differentiated upon long-term exposure to G5 exhibit a time-dependent expression of background membrane currents with differential pharmacological sensitivity.

### Identification of the ion currents expressed by G5-treated primary cortical astrocytes

Since 12–16 DOT G5 astrocytes display an increase in K^+^ conductance, we next sought to identify the underlying channels. Cortical astrocytes in vitro express Kir-channel-mediated currents under specific culture conditions [2, 21] and two-p-domain K^+^ (K2P) channels that are activated by specific stimuli [22, 25, 51]. In vivo both channels contribute to the large background conductance [64, 94]. However, the high Ba^2+^ sensitivity of the K^+^ conductance expressed by astrocytes upon G5 for 12–16 DOT diminished the probability of the contribution of K2P-mediated current because in cultured astrocytes, K2P channels are only slightly inhibited by higher Ba^2+^ concentration [22]. We hence hypothesized that the G5-induced K^+^ conductance was mediated by Kir channels.

**Fig. 5:**
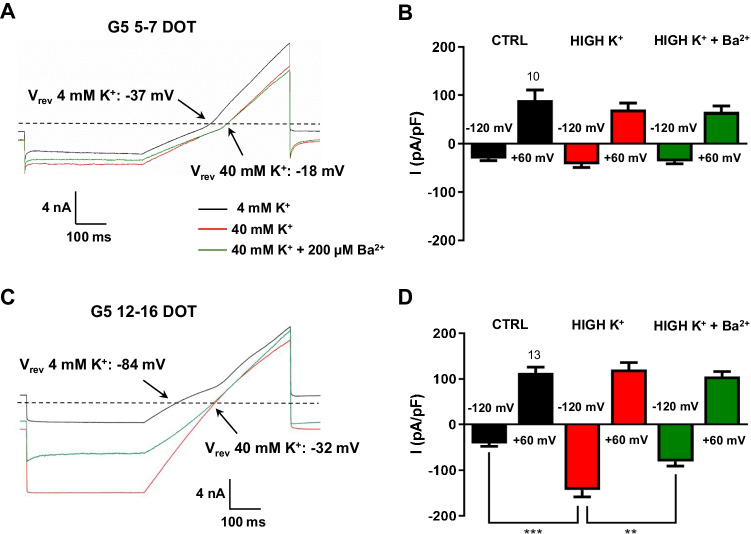
Identification of a potassium inward rectifier as mediator of the barium-sensitive current expressed by G5-treated primary cortical astrocytes. **A** Representative ramp currents elicited with a stimulation protocol as in Fig. 3 in a 5–7 DOT G5 astrocyte in extracellular 4 mM K^+^ (black trace), after increasing K^+^ to 40 mM by isotonic replacement of sodium (red trace) and following the subsequent application of Ba^2+^ (green trace). The ten-fold increase in K^+^ did not modify the current amplitude and kinetics both in the absence and presence of Ba^2+^ but caused a ~20 mV depolarized shift of the current reversal potential (*V*_rev_). Dashed line is the zero-current level. **B** Bar graph of current densities at +60 mV and −120 mV in 4 mM extracellular K^+^ (ctrl), upon 40 mM K^+^ before (high K^+^) and following the maximal effect of Ba^2+^ administration in 40 mM K^+^ (high K^+^ + Ba^2+^) in 5–7 G5 astrocytes. **C** Representative currents evoked in a 12–16 DOT G5 astrocyte with the same protocol as in A. The ten-fold increase in extracellular K^+^ induced a ~50 mV positive shift in current reversal potential, an increase in membrane conductance at ramp potentials more negative than the reversal potential, and the partial depression by Ba^2+^ administration. Dashed line is the zero-current level. **D** The quantitative analysis shows that the ten-fold increase in extracellular K^+^ enhanced the current magnitude only at −120 mV, which was partially attenuated by Ba^2+^ administration. **p<0.01 and ***p<0.001 with one-way ANOVA followed by Bonferroni’s multiple comparisons test. Numbers above black bars in B and D depict the number of cells in each condition

In cultured astrocytes, the kinetics of Kir current depend on extracellular K^+^ concentration ([K^+^]_o_) that yields an increase in chord conductance at potentials more negative than *V*_rev_ [2, 71]. We analyzed the ramp current kinetics in physiological [K^+^]_o_ and upon a tenfold increase. The results show that at 5–7 DOT upon G5, the change in [K^+^]_o_ from 4 to 40 mM caused a positive shift of ~ 20 mV in *V*_rev_ but did not affect the chord conductance (Fig. 5A, B). By contrast, at 12–16 DOT with G5, the same experimental protocol induced a ~ 50 mV positive shift of *V*_rev_ and an increase in Ba^2+^-sensitive chord conductance at potentials negative to *V*_rev_ (Fig. 5C, D). These results support the view that cultured astrocytes grown for long-term in G5-containing medium express a K^+^ current mediated by Kir channels that, however, becomes clearly apparent only after exposure to G5 for more than 5–7 DOT.

**Fig. 6:**
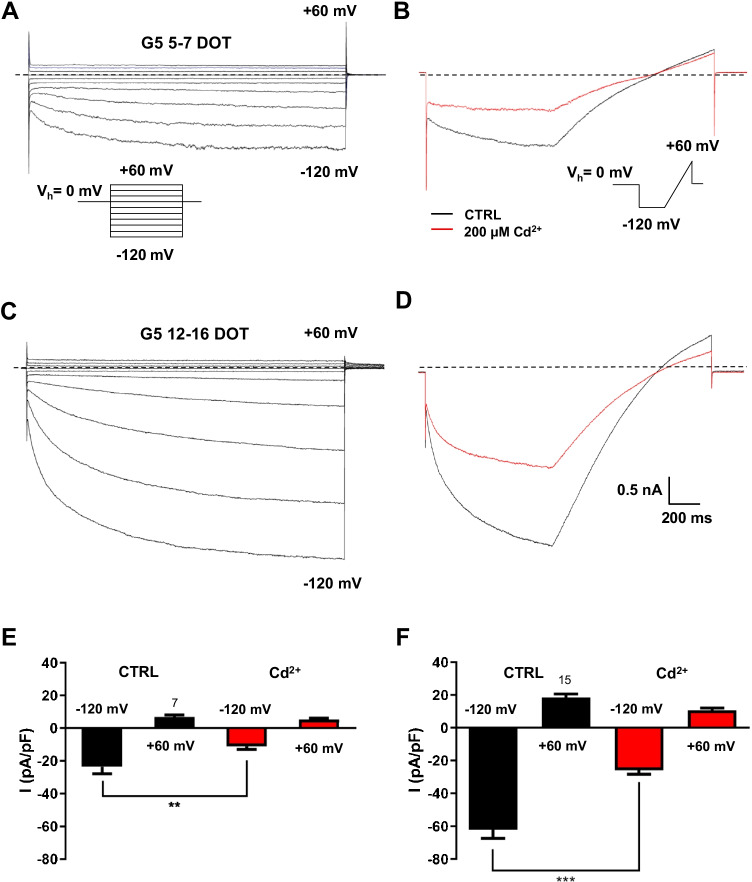
An inward rectifier Cl^−^ current underlies the cadmium-sensitive conductance expressed by G5-treated primary cortical astrocytes.**A **Representative family of currents elicited in a 5–7 DOT G5 astrocyte with a voltage steps protocol (inset) consisting of 2-s long voltage steps from a *V*_*h*_ of 0 mV applying step potentials from −120 to +60 mV in 20 mV increments and measured using intra- and extra-cellular solutions with symmetrical high chloride (Cl^−^) and the monovalent cations replaced with cesium. Time-dependent currents activated slowly and were strongly inwardly rectifying. **B** The slow ramp current (180 mV/1 s) activated following a voltage step of 800 ms to −120 mV from a *V*_*h*_ of 0 mV (inset) in the same astrocyte of A was markedly depressed by extracellular Cd^2+^ (200 µM, red trace), which inhibits inward rectifier Cl^−^ channels. **C** Representative voltage-step currents in a 12–16 DOT G5 astrocyte recorded in the same conditions as in A are grater. **D** The ramp current in the same astrocyte retained the partial inhibition by Cd^2+^. Scale bars are the same for all current traces. Dashed lines represent the zero-current level. **E**–**F** Bar graph of ramp current densities of the inward rectifier Cl^−^ current at +60 and −120 mV measured in astrocytes exposed to G5 for 5–7 DOT (**E**) and 12-16 DOT (**F**) in the absence (ctrl) and following the maximal inhibition upon extracellular administration of Cd^2+^ (200 µM). **p<0.01 and ***p<0.001 with paired Student’s *t*-test. Numbers above bars depict the number of cells in each condition

We next attempted to identify the membrane conductance insensitive to Ba^2+^ and attenuated by Cd^2+^, which was activated at hyperpolarized potentials in 5–7 DOT G5 astrocytes. There is evidence that in addition to Kir channels, both in vitro and in vivo astrocytes express two types of hyperpolarization-activated currents mediated by anion and cation channels [20, 21, 28, 45, 46, 68]. To rule out the contribution of the cationic current mediated by hyperpolarization‐activated cyclic nucleotide‐gated (HCN) channels expressed by reactive astrocytes in vivo [32], experiments were performed by replacing intra- and extracellular Na^+^ and K^+^ with cesium (Cs^+^), which inhibits HCN [52] and with symmetrical high Cl^−^. Under these ionic conditions, 5–7 DOT G5 astrocytes exhibited large strongly inward-rectifying currents which activated slowly at step potentials more negative than –20 mV and did not show voltage dependent inactivation (Fig. 6A). The hyperpolarization-activated conductance was significantly attenuated by submillimolar Cd^2+^ (Fig. 6B, E) corroborating the tenet that it was mediated by the inwardly rectifying Cl^−^ current previously identified in cultured astrocytes [20, 21, 45]. In 12–16 DOT G5 astrocytes, the amplitude of the Cl^−^ conductance was larger (Fig. 6C, D) owing to an increase in current density (Fig. 6F).

Altogether, these results indicate that the morphological differentiation of long-term G5-treated astrocytes is accompanied by the early expression of an inward rectifier Cl^−^ current, which precedes the functional appearance of the inward rectifier K^+^ current.

### Molecular identification of the channel proteins mediating the increased membrane conductance in G5-treated primary cortical astrocytes

**Fig. 7:**
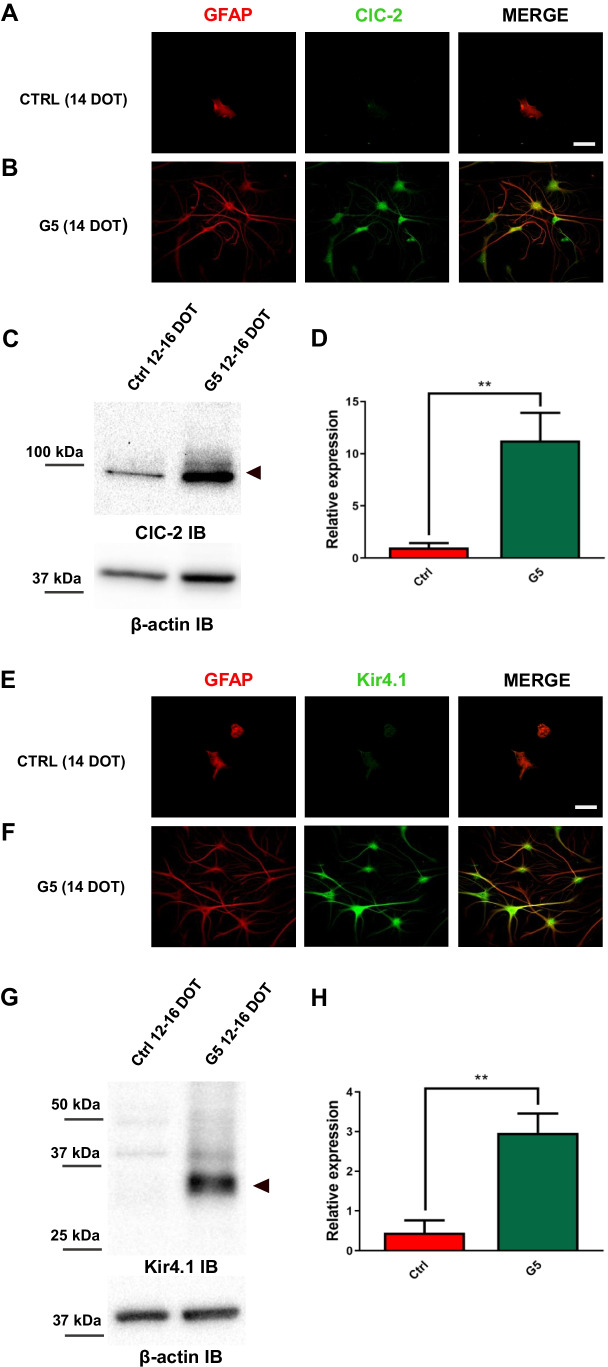
Variations in molecular expression of chloride and potassium channels in G5-treated primary cortical astrocytes.**A**–**B **Confocal analyses on sub-confluent astrocytes co-immunostained for glial fibrillary acidic protein (GFAP, red) and ClC-2 protein (green). ClC-2 is undetectable in 14 DOT untreated GFAP-positive astrocytes (**A**) but is present in 14 DOT G5 astrocytes (**B**). Merge image depicts partial signal overlay. **C**–**D** Representative western blot (**C**) of ClC-2 monomer from total lysates of untreated (ctrl) and 12-16 DOT G5 astrocytes with relative densitometric analysis (**D**, *n*=3).**E**–**F **Confocal analyses performed on sub-confluent astrocytes co-immunostained for GFAP (red) and Kir4.1 (green). Kir4.1 staining is negligible in untreated GFAP-positive astrocytes (ctrl, **E**) and strongly upregulated in 14 DOT G5 astrocytes (**F**). Merge image depicts partial signal overlay. Scale bars in A and E: 20 µm. **G**–**H** Representative western blot (**G**) of Kir4.1 monomer from total lysates of untreated (ctrl) and 12–16 DOT G5-treated astrocytes with relative densitometric analysis (**H**, *n*=3). **p<0.01 with unpaired Student’s *t*-test

The functional analyses supported the view that astrocytes morphologically differentiated upon long-term G5 exposure express inward rectifier Cl^−^ and K^+^ currents. To determine whether these results were associated with an increment in ion-channel protein synthesis, we performed immunocytochemical and immunoblotting analyses in 12–16 DOT astrocytes in the absence and presence of G5. The functional analysis suggested that the increase in Cl^−^ conductance was mediated by the upregulation of ClC-2, which was shown to be functionally expressed in astroglial cells both in vitro and in vivo[36, 46]. The results obtained by confocal analysis in untreated (ctrl) and G5-treated sub-confluent astrocytes at 14 DOT show that ClC-2 (green) was highly expressed both in cell body and in multi-branched processes of GFAP-positive cells (red) but was virtually undetectable in untreated astrocytes (Fig. 7A, B). Immunoblotting analysis revealed that in lysates of long-term G5-treated astrocytes, the expression level of ClC-2 monomer was tenfold higher compared to that of untreated cells (Fig. 7C, D). Since the ClC-2-mediated currents developed early upon G5 treatment, we also assessed the protein expression in whole-cell lysates of astrocytes at 5–7 DOT. Surprisingly, we did not observe any difference in total protein expression between untreated astrocytes and those G5-treated (Fig. S3).

We next characterized the expression pattern of the Kir4.1 channel, which previous studies identified to be the predominant Kir channel protein expressed in astrocytes from different regions in vivo [29, 31, 42, 60, 75] but also under some culture conditions in vitro [2]. The immunofluorescence analysis of untreated (ctrl) and 14 DOT G5-treated astrocytes revealed that in GFAP positive cells (red), K_ir_4.1 expression (green) was not detectable in control astrocytes but was diffusely present in G5-treated astrocytes (Fig. 7E, F). Qualitatively similar results were obtained by comparing the immunoblot determinations in the two conditions which show that in whole-cell protein extracts, the monomeric form of Kir4.1 was increased by ~ sevenfold (Fig. 7G, H).

### G5-treated primary cortical astrocytes display a non-reactive biochemical phenotype

**Fig. 8:**
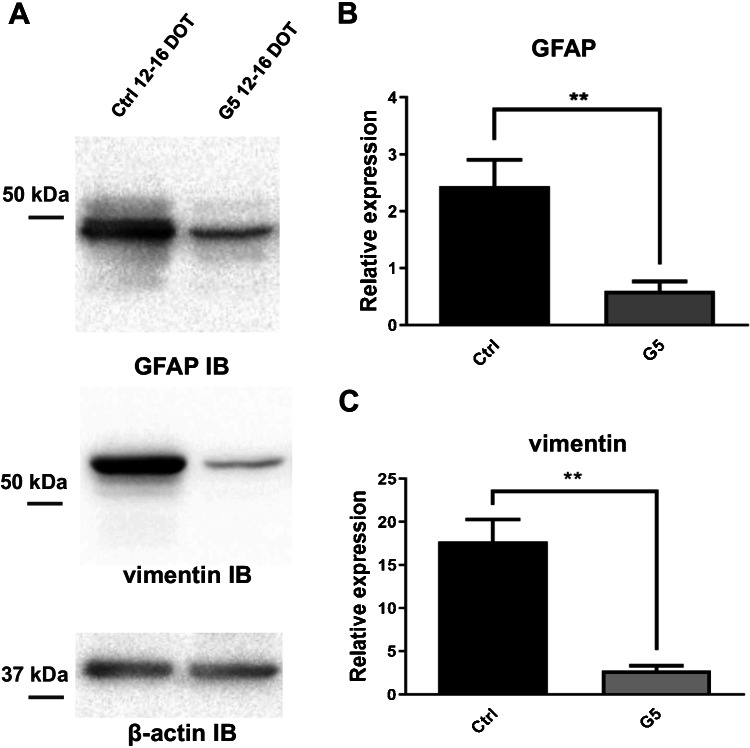
Changes in expression of intermediate filament proteins in G5-treated primary cortical astrocytes.**A**)Representative western blot of GFAP and vimentin from total lysates of untreated (ctrl) and 12-16 DOT G5 astrocytes. **B**-**C**) The densitometric analyses (*n*=3 for each condition) show the marked diminution in GFAP and vimentin levels upon prolonged G5 treatment. ** p<0.01 with unpaired Student’s *t*-test

Since changes in morphology denoted by the appearance of process elongation is also observed in reactive astrocytes [18], we evaluated whether the morphological differentiation induced by G5 challenge was associated to an increase in GFAP expression, which is a typical hallmark of reactive astrocytes [17]. The immunoblot analysis shows that compared to untreated astrocytes, those exposed to G5 for 12–16 DOT exhibited a robust diminution of GFAP expression (Fig. 8A, B), and the same result was observed for the other intermediate filament vimentin (Fig. 8A, C), which is also upregulated in reactive astrocytes [66]. Altogether, this result suggests that the long-term culturing of primary rat cortical astrocytes in G5-containing chemically defined medium promotes the growth of non-reactive, quiescent astrocytes that display functional features observed in astrocytes in vivo.

## Discussion

The main objective of this study was to determine the long-term functional consequences of the grown of primary cortical astrocytes in a chemical-defined medium supplemented with a cocktail (G5) of growth factors and hormones that previous studies reported to cause their in vitro activation and morphological differentiation [8, 27, 86, 87]. We show that the long-term G5 challenge caused the gradual, time-dependent morphological differentiation of the astrocytes that acquire a multi-branched, process-bearing phenotype accompanied by variations of passive membrane properties and by time-dependent differential expression of K^+^ and Cl^−^ membrane channels involved in the homeostatic functions of astrocytes.

Usually, cultured astrocytes are grown in a medium containing 10% FBS which allows rapid proliferation [47]. At confluence FBS-grown cortical astrocyte monolayer has a cobblestone phenotype, and re-plated isolated astrocytes display a fibroblast-like, epithelioid morphological phenotype [54]. In our experimental model, FBS was gradually omitted when astrocytes reached confluence (~ 10 days), and re-plated astrocytes in the absence of FBS were supplemented with G5 for up to 2 weeks thereafter. During this period, astrocytes gradually changed their shape displaying polarized processes departing from the cell body after 5–7 DOT and acquiring a multipolar, fine-branched phenotype with a retracted cell body at 12–16 DOT. These morphological alterations resemble those observed in long-term cultured astrocytes grown in various chemically defined medium [54]. Similar results have also been obtained with a method in which immunopanning of microglia, macroglia, and neurons was used to culture astrocytes in FBS-free, heparin-binding epidermal growth factor (EGF)-containing medium [23, 92]. However, it is worth noting that also in those approaches, FBS was used in some purification steps of the astrocyte cultures. Recently, a method using a FBS-free, chemically defined medium containing fibroblast growth factor-2 (FGF2) and epidermal growth factor (EGF) was shown to promote the growth of mice-cultured astrocytes with morphological and biosynthetic features similar to those of in vivo astrocytes and characterized, compared to untreated cells, by a process-bearing morphology and an increase of glutamate uptake [70]. Of note, even though FGF and EGF are present in G5 cocktail, compared to untreated astrocytes, 12–16 DOT G5 astrocytes displayed a slight decrease in expression of the principal glutamate transporter GLT-1 (Fig. S4). Whether this negative modulation is due to the cellular morphological rearrangement or an antagonistic effect of other components of G5 administered in the absence of FBS remains to be established.

Our study clearly shows that the morphological differentiation of G5-treated cortical astrocytes was paralleled by variations in membrane conductance. The results indicate that G5 challenge for 5–7 DOT caused a marked decrease in membrane resistance, which, however, compared to untreated astrocytes, was not accompanied by a significant change in RMP. The electrophysiological and pharmacological analyses revealed that the increase in membrane conductance was due, at least in part, to an increase in functional expression of the Cl^−^ channel ClC-2, which started early during G5 treatment and increased throughout the whole period of exposure. Notably, the upregulation of ClC-2-mediated Cl^−^ inward rectifier at 5–7 DOT was not paralleled by an increment in total protein level. This discrepancy could be due to a G5-mediated augment in trafficking of ClC-2 from a cytosolic pool to the plasma membrane. In this context, previous work in cultured astrocytes identified significant amount of ClC-2 in the Golgi complex [78]. Moreover, cell surface expression and function of ClC-2 was shown to be upregulated by microtubule perturbation [14]. Whether G5 treatment induces the early effect on ClC-2-mediated conductance through changes in microtubule-associated proteins that regulate astrocyte differentiation [13] remains to be established. At variance, G5 challenge for 12–16 DOT also upregulated newly synthesized ClC-2 proteins, a result that was mirrored by a further increase in Cl^−^ inward rectifier.

ClC-2 is a member of the large family of Cl^−^ channels that in mammals consist of 9 subtypes [35]. ClC-2 is expressed in various cell preparations and is highly enriched in the brain [10, 81]. It is inhibited by submillimolar concentrations of Cd^2+^ and Zn^2+^ and allows Cl^−^ outflow at membrane potentials below the equilibrium potential for Cl^−^ [37]. Astrocytes express a variety of Cl^−^ channels [16, 87]. ClC-2 was shown to be expressed in astrocytes both in vitro and in situ [36, 46, 77]. In brain slices, ClC-2 is localized at terminals of astrocyte processes abutting GABAergic synapses in the hippocampus [77]. An inward rectifier Cl^−^ current with pharmacological and biophysical properties identical to ClC-2 was reported to be expressed in cultured rat cortical astrocytes long-term treated with dBcAMP to induce their morphological differentiation [20, 21] and in astrocytes co-cultured with neurons [45]. The result that the functional expression of the inward rectifier Cl^−^ conductance mediated by ClC-2 is an early event associated to the morphological differentiation of the primary astrocytes induced by G5 exposure raises the question of whether this channel could play a role in the regulation of the differentiation process. Interestingly, in situ ClC-2 is absent in morphologically immature astrocytes and in astrocytes that display morphological alterations within a brain lesion [46]. However, since reverse causality cannot be ruled out, further studies are warranted to address this issue.

The role of ClC-2 in astrocytes as well as in other cell types is still largely elusive [34]. Genetic ablation of ClC-2 did not cause overt defects in brain structural organization except widespread vacuolization of the white matter and spinal cord typical of leukoencephalopathy [4]. The absence of ClC-2 has been associated with progressive neurodegeneration in old mice a result that was interpreted as due to astrocyte activation leading to defect in neurotransmission [12]. Recent works in cultured astrocytes indicate that ClC-2 assembles with auxiliary subunits that change its biophysical properties [36, 78]. Those findings suggested that ClC-2 also might mediate Cl^−^ influx necessary to compensate the K^+^ dynamics. Overall, the results of this study indicate that G5-treated cultured astrocytes could be a suitable experimental model for getting more insights on the functional impact of ClC-2 in astrocyte biology.

Our data also show that the ohmic profile of the large membrane conductance of cortical astrocytes exposed to G5 for 12–16 DOT is partially mediated by the upregulation of the inward rectifier K^+^ channel Kir4.1. Kir4.1 is a member of the large family of Kir channels composed of 15 subtypes [30]. It is also the major component of the large astrocytic K^+^ conductance and promotes the very negative RMP of astrocytes in vivo [15, 38, 60, 71, 75]. Kir4.1 is crucially involved in the regulation of [K^+^]_o_ [9, 15, 39] and to augmenting the efficacy of glutamate uptake [15, 33]. In cultured astroglia, the evidence are more contradictory with reports indicating the presence of Kir4.1 [39, 60] and others failing to detect its functional expression [3, 21, 79]. These discrepancies could be related to the culture conditions and/or the region of the CNS from which the tissues to prepare the culture were obtained. In this respect, it is well known that in situ Kir4.1 expression is heterogeneous throughout brain regions and in the spinal cord [53, 57, 59, 69, 80]. The current study confirms that under our experimental conditions and in the absence of G5, cultured cortical astrocytes do not display significant Kir4.1-mediated current. Even at 16 DOT, only outward rectifier K^+^ current insensible to bath application of submillimolar Ba^2+^ could be detected in untreated astrocytes. By contrast in astrocytes at 8–11 DOT with G5, there was an increase in the proportion of astrocytes with a very negative RMP and an overall decrease in input resistance with a further decrement at 12–16 DOT. These results, together with the marked depression of the negative currents by Ba^2+^ at a concentration that selectively inhibits Kir4.1, suggest that G5 exposure promotes the gradual functional appearance of Kir4.1. This finding was corroborated by the observation that G5 challenge for 12–16 DOT induced a strong increase in Kir4.1 protein expression when compared to untreated cultures. Of note, compared to untreated astrocytes, the challenge with G5 at 5–7 and 12–16 DOT also caused an increment of positive current activated at depolarized membrane potentials. The increase in K^+^ outflow through Kir4.1 could only partially explain the increase in positive current at 12–16 DOT because the upregulation was already observed in astrocytes at 5–7 DOT when Kir4.1 was not functionally expressed and Ba^2+^ application had minimal effect on outward current. However, the observation that this positive conductance was totally abolished when intracellular K^+^ was replaced with the K^+^-channel impermeant cation Cs^+^ suggests that it was mediated by an increase in K^+^ current. Noteworthy, the upregulation of outward K^+^ conductance could contribute to the consistent negative RMP observed in 5–7 G5 astrocytes in the presence of the depolarizing effect of the Cl^−^ inward rectifier under our experimental conditions.

It has been reported that G5 challenge also promotes an increase saxitoxin-sensitive voltage-gated Na^+^ channels [90]. The results of previous research [21] and of the current study do not support the presence of voltage-dependent Na^+^ currents in untreated astrocytes. However, whether G5 affects the transcripts and/or whole-cell protein levels of the various Na^+^ channels isoforms identified in different astrocyte preparations remains to be established [63].

Previous works showed that cultured astrocytes challenged with G5 displayed an increment of glutamate uptake capability linked to the increased expression of glutamate transporters [84–86]. However, whether that effect is influenced by additional mechanisms is unknown. The functional upregulation of Kir4.1 could partially explain the rise in glutamate uptake observed in G5-treated cultured astrocytes. A direct correlation between Kir4.1 expression and glutamate uptake has been demonstrated as in vivo the genetic ablation of Kir4.1 is associated to a decrement in glutamate uptake mediated by the Na^+^-dependent cotransporter GLT-1 [15]. Similar results have been described in cultured astrocytes in which Kir4.1 expression was downregulated by RNA interference [39]. It remains to be ascertained the effect on glutamate uptake of the large increase in Kir4.1 current in long-term G5-treated cortical astrocytes particularly in the context of the concomitant small diminution in total expression of GLT-1 transporter observed under these conditions. Whether the increase in expression of the Cl^−^ channel ClC-2 plays a role in the regulation of glutamate uptake also warrants further investigations.

Primary cultured astrocytes have been often used for studying the astrocyte properties in physiological as well as pathological contexts [40, 49]. We and others have previously shown that incubation of cultured cortical astrocytes with the cell permeable cAMP analog dBcAMP caused morphological and biochemical changes [21, 43, 76, 89]. The adherence of that culture model to physiological or pathological conditions in vivo is still uncertain [19, 62, 88], and therefore, better in vitro models are required. The results of this study support the view that the long-term astrocyte culturing in a chemically defined medium containing G5 in the absence of FBS produces changes in astrocyte morphology and the acquisition of a quiescent phenotype since 12–16 DOT with G5 caused a strong downregulation in the expression of the intermediate filaments GFAP and vimentin, which are upregulated in reactive astrocytosis in vivo [41, 65, 73]. This observation contrasts with previous work in which few days of G5 challenge caused the upregulation of GFAP and vimentin [27]. The reason for this discrepancy is unclear but the fact that in our study G5 challenge was performed in the absence of FBS could play a role. To corroborate this hypothesis, it was previously reported that FBS affects several properties of cultured astrocytes [11]. Moreover, the fact that the G5 challenge was prolonged up to 2 weeks could also be a plausible explanation. Altogether, these results indicate that a supplement containing growth factors and hormones such as those present in G5 cocktail supports the long-term growth of primary cultured astrocytes in the absence of FBS and induce their morphological and functional differentiation by upregulating relevant homeostatic channel proteins.

## Conclusions

Even though major advances in the understanding about the role of astrocytes in the physiology and pathophysiology of the CNS has been obtained from studies performed in primary cultured astrocytes, in the last decades, it has become clear that in vitro models not always mimic perfectly the complex situation occurring in vivo [40]. Despite this evidence, the use of a reductionist model such as primary cultures is still essential to address the contribution of specific stimuli in determining the dynamics of astrocyte plasticity under controlled conditions. In this context in the last years, great effort has been made to define conditions that more closely resembles those in vivo [23, 74]*.*The results of this study add another piece of evidence to the view that the manipulation of culture conditions through the addition of specific growth factors and hormones could be used to create a valid in vitro platform to investigate the modulatory processes of homeostatic functions dependent on the expression of plasma membrane ion channels.

## Supplementary Information

Below is the link to the electronic supplementary material.

Supplementary file1 (PPTX 1.36 MB)
