# Supplementary material for: Dynamic expression of homeostatic ion channels in differentiated cortical astrocytes in vitro
Source: Pflugers Arch. 2021 Nov 4;474(2):243–60. doi: 10.1007/s00424-021-02627-x (PMC8766406; doi:10.1007/s00424-021-02627-x)
Supplement: Supplementary file 1 — Supplementary file1 (PPTX 1.36 MB) [file 424_2021_2627_MOESM1_ESM.pptx]

## Slide 1
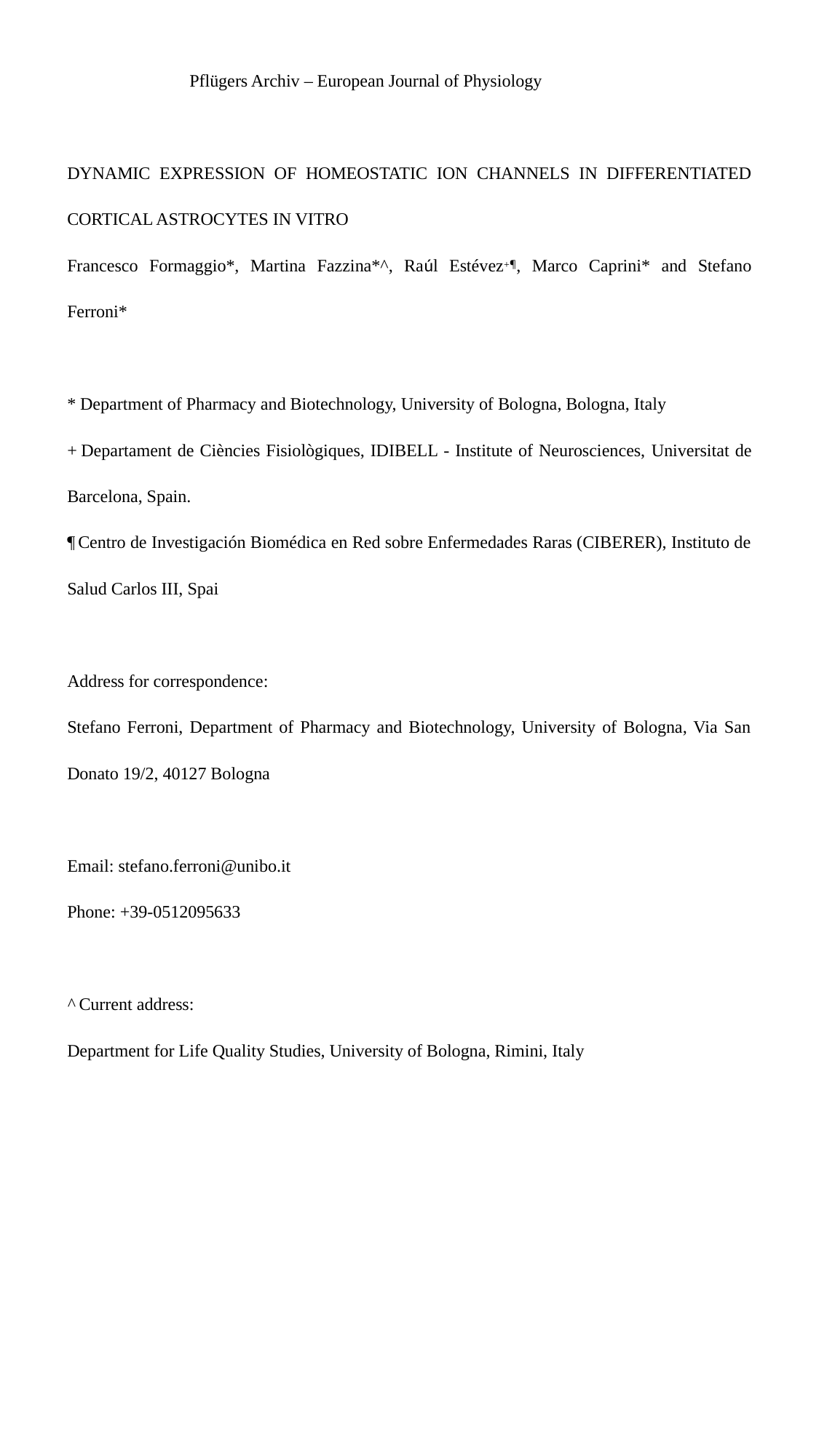

Pflügers Archiv – European Journal of Physiology
DYNAMIC EXPRESSION OF HOMEOSTATIC ION CHANNELS IN DIFFERENTIATED CORTICAL ASTROCYTES IN VITRO
Francesco Formaggio*, Martina Fazzina*^, Raúl Estévez+¶, Marco Caprini* and Stefano Ferroni*
* Department of Pharmacy and Biotechnology, University of Bologna, Bologna, Italy
+ Departament de Ciències Fisiològiques, IDIBELL - Institute of Neurosciences, Universitat de Barcelona, Spain.
¶ Centro de Investigación Biomédica en Red sobre Enfermedades Raras (CIBERER), Instituto de Salud Carlos III, Spai
Address for correspondence:
Stefano Ferroni, Department of Pharmacy and Biotechnology, University of Bologna, Via San Donato 19/2, 40127 Bologna
Email: stefano.ferroni@unibo.it
Phone: +39-0512095633
^ Current address:
Department for Life Quality Studies, University of Bologna, Rimini, Italy

## Slide 2
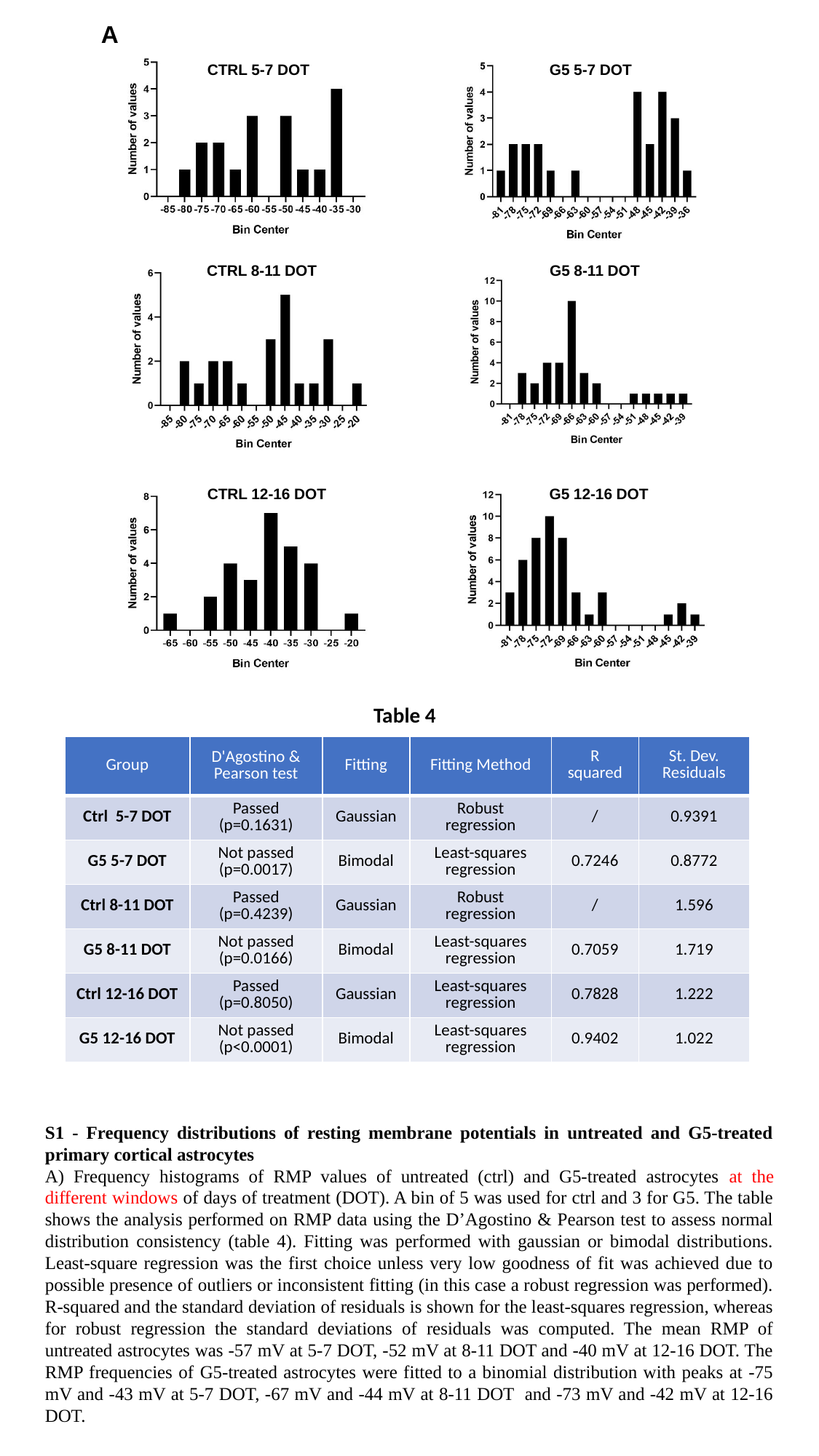

A
CTRL 5-7 DOT
G5 5-7 DOT
G5 8-11 DOT
CTRL 8-11 DOT
CTRL 12-16 DOT
G5 12-16 DOT
Table 4
| Group | D'Agostino & Pearson test | Fitting | Fitting Method | R squared | St. Dev. Residuals |
| --- | --- | --- | --- | --- | --- |
| Ctrl 5-7 DOT | Passed (p=0.1631) | Gaussian | Robust regression | / | 0.9391 |
| G5 5-7 DOT | Not passed (p=0.0017) | Bimodal | Least-squares regression | 0.7246 | 0.8772 |
| Ctrl 8-11 DOT | Passed (p=0.4239) | Gaussian | Robust regression | / | 1.596 |
| G5 8-11 DOT | Not passed (p=0.0166) | Bimodal | Least-squares regression | 0.7059 | 1.719 |
| Ctrl 12-16 DOT | Passed (p=0.8050) | Gaussian | Least-squares regression | 0.7828 | 1.222 |
| G5 12-16 DOT | Not passed (p<0.0001) | Bimodal | Least-squares regression | 0.9402 | 1.022 |
S1 - Frequency distributions of resting membrane potentials in untreated and G5-treated primary cortical astrocytes
A) Frequency histograms of RMP values of untreated (ctrl) and G5-treated astrocytes at the different windows of days of treatment (DOT). A bin of 5 was used for ctrl and 3 for G5. The table shows the analysis performed on RMP data using the D’Agostino & Pearson test to assess normal distribution consistency (table 4). Fitting was performed with gaussian or bimodal distributions. Least-square regression was the first choice unless very low goodness of fit was achieved due to possible presence of outliers or inconsistent fitting (in this case a robust regression was performed). R-squared and the standard deviation of residuals is shown for the least-squares regression, whereas for robust regression the standard deviations of residuals was computed. The mean RMP of untreated astrocytes was -57 mV at 5-7 DOT, -52 mV at 8-11 DOT and -40 mV at 12-16 DOT. The RMP frequencies of G5-treated astrocytes were fitted to a binomial distribution with peaks at -75 mV and -43 mV at 5-7 DOT, -67 mV and -44 mV at 8-11 DOT and -73 mV and -42 mV at 12-16 DOT.

## Slide 3
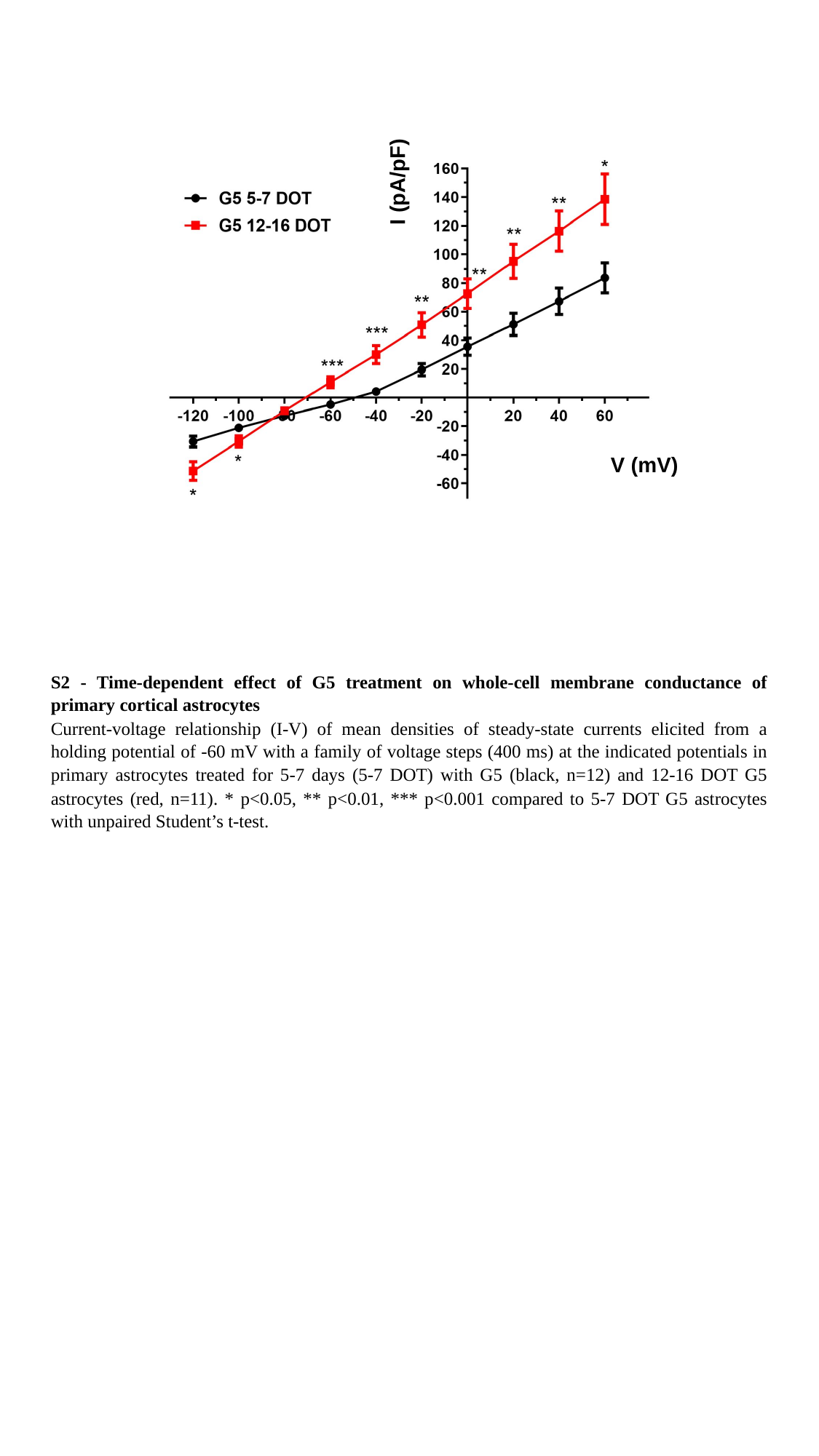

I (pA/pF)
 V (mV)
S2 - Time-dependent effect of G5 treatment on whole-cell membrane conductance of primary cortical astrocytes
Current-voltage relationship (I-V) of mean densities of steady-state currents elicited from a holding potential of -60 mV with a family of voltage steps (400 ms) at the indicated potentials in primary astrocytes treated for 5-7 days (5-7 DOT) with G5 (black, n=12) and 12-16 DOT G5 astrocytes (red, n=11). * p<0.05, ** p<0.01, *** p<0.001 compared to 5-7 DOT G5 astrocytes with unpaired Student’s t-test.

## Slide 4
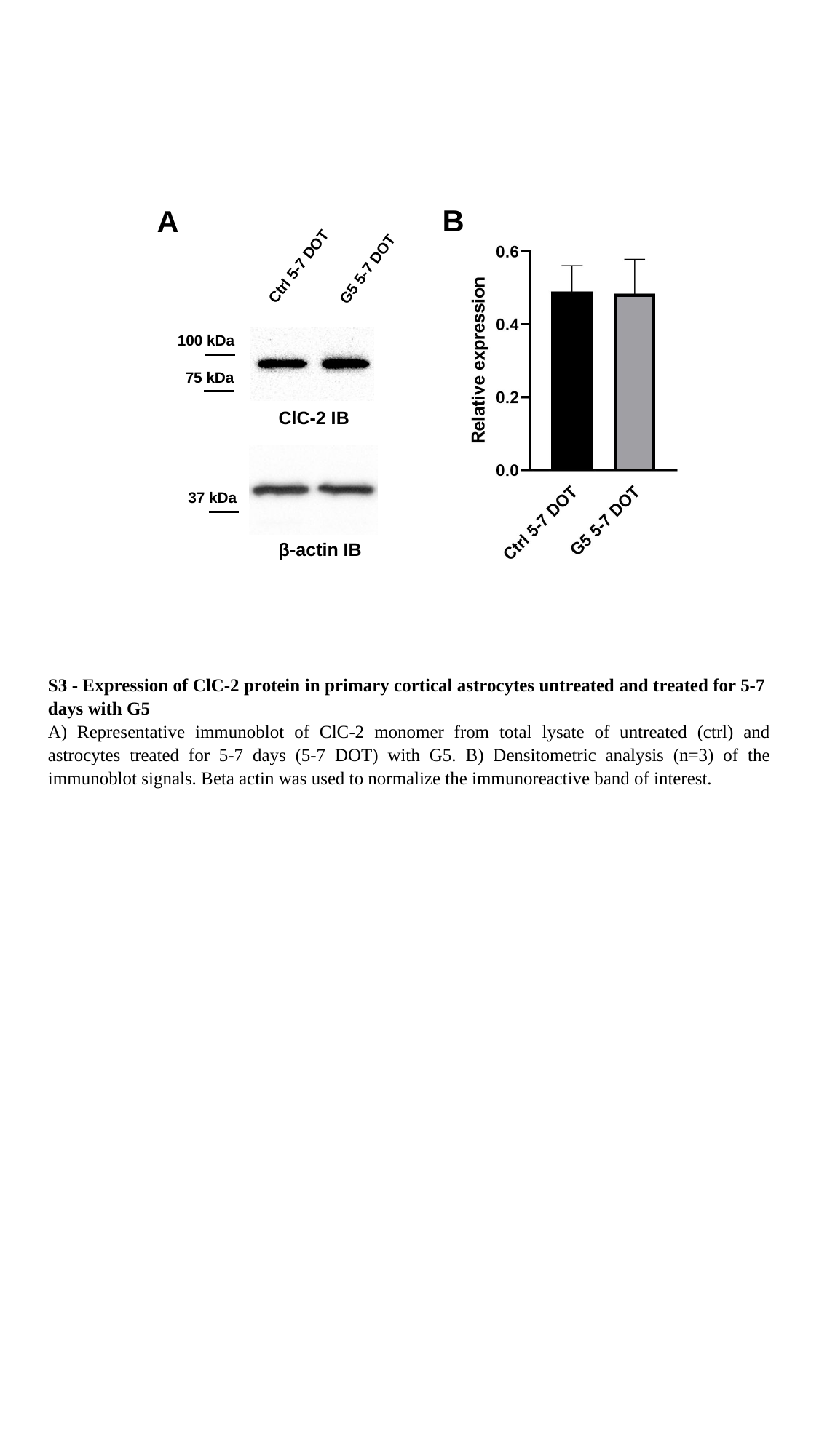

B
A
Ctrl 5-7 DOT
G5 5-7 DOT
100 kDa
75 kDa
ClC-2 IB
37 kDa
β-actin IB
S3 - Expression of ClC-2 protein in primary cortical astrocytes untreated and treated for 5-7 days with G5
A) Representative immunoblot of ClC-2 monomer from total lysate of untreated (ctrl) and astrocytes treated for 5-7 days (5-7 DOT) with G5. B) Densitometric analysis (n=3) of the immunoblot signals. Beta actin was used to normalize the immunoreactive band of interest.

## Slide 5
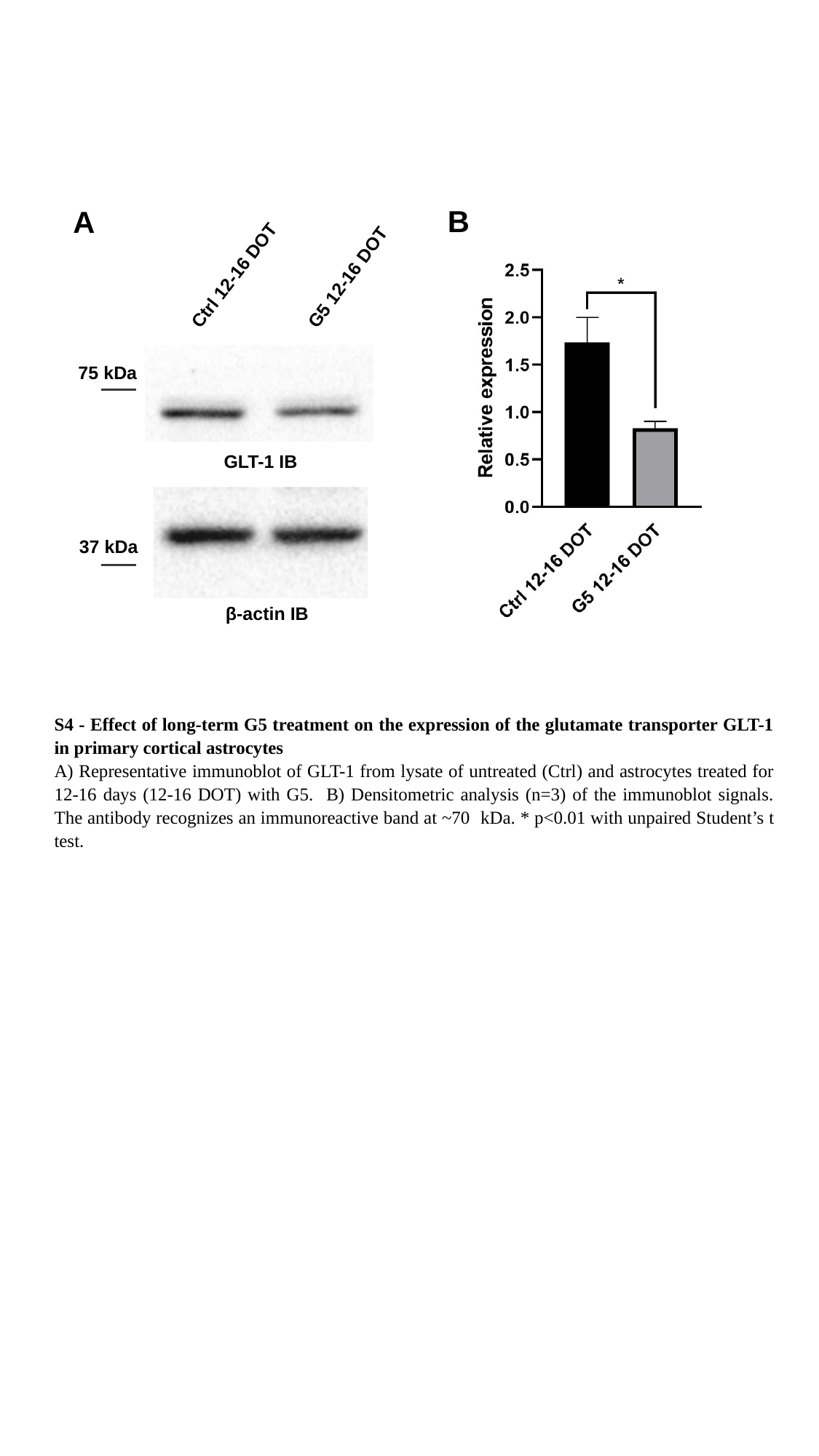

B
A
Ctrl 12-16 DOT
G5 12-16 DOT
75 kDa
GLT-1 IB
37 kDa
 β-actin IB
S4 - Effect of long-term G5 treatment on the expression of the glutamate transporter GLT-1 in primary cortical astrocytes
A) Representative immunoblot of GLT-1 from lysate of untreated (Ctrl) and astrocytes treated for 12-16 days (12-16 DOT) with G5. B) Densitometric analysis (n=3) of the immunoblot signals. The antibody recognizes an immunoreactive band at ~70 kDa. * p<0.01 with unpaired Student’s t test.
